# Effects of Coal and Sewage Sludge Ashes on Macronutrient Content in Maize (*Zea mays* L.) Grown on Soil Contaminated with Eco-Diesel Oil

**DOI:** 10.3390/ma15020525

**Published:** 2022-01-11

**Authors:** Mirosław Wyszkowski, Jadwiga Wyszkowska, Natalia Kordala, Agata Borowik

**Affiliations:** 1 Department of Agricultural and Environmental Chemistry, University of Warmia and Mazury in Olsztyn, Łódzki 4 Sq., 10-727 Olsztyn, Poland; natalia.kordala@uwm.edu.pl; 2 Department of Soil Science and Microbiology, University of Warmia and Mazury in Olsztyn, Łódzki 3 Sq., 10-727 Olsztyn, Poland; agata.borowik@uwm.edu.pl

**Keywords:** Eco-Diesel oil, ash, macronutrients in *Zea mays* L.

## Abstract

Petroleum hydrocarbons, as aggressive components of diesel oils, after migration to the land environment can alter the activity and efficiency of ecosystems. They can also be dangerous to animal and human health. Eco-friendly methods for the reclamation of affected soils is necessary to manage degraded lands. One such method is the use of ashes. The aim of this research was to determine how soil pollution with diesel oil (brand name, Eco-Diesel) affects the chemical composition of maize (*Zea mays* L.) and whether the application of ash from a combined heat and power plant, as well as from sewage sludge incineration, could reduce the potentially adverse impact of diesel oil on plants. The research results demonstrated that soil contamination with Eco-Diesel oil modified the content of selected macronutrients in the analyzed crop plant. Eco-Diesel oil had a negative effect on maize yield. The highest diesel oil dose in a series without neutralizing substances had a positive effect on the accumulation of most elements, except nitrogen and sodium. Soil enrichment with ash differentiated the content of macronutrients, mainly nitrogen and phosphorus, in the aerial biomass of maize. The ashes increased the yield of maize and content of some macronutrients, mainly nitrogen but also calcium, the latter in a series where soil was treated with ash from sewage sludge thermal recycling. Both types of ash also resulted in a decrease in the plant content of phosphorus, while ash from hard coal caused a slight reduction in the content of potassium in maize. Ash of different origins can be an effective solution in the reclamation of degraded soils, which may then be used for growing energy crops.

## 1. Introduction

Maize is a plant species with considerable economic importance and versatile use. Its biomass can serve for generation of energy through direct incineration or be processed to produce liquid and gas fuels [1,2]. The high yielding potential of this plant, up to 30–50 Mg (megagrams) per 1 hectare [3], has stimulated interest in using maize in biochemical processes, i.e., biogas production (fresh mass, silage) or bioethanol production (grain) [4], but also in direct incineration and thermo-chemical processes, such as pyrolysis or gasification employed to produce methanol, biogas and pyrolytic oils [1]. The high calorific value of the biomass, in the range of approximately 15.5 to 18.1 MJ kg^−1^, is an additional argument for using maize for energy purposes [3,5,6].

Use of plant biomass is one of the ways to achieve diversification in sources of energy and engine fuels, thereby reducing the negative impact of burning fossil fuels and economic development on the environment [7,8]. As well as being the main source of energy in many areas of communal life, crude oil is fundamental to the efficient functioning of economies around the world. The release of oil derivatives, both incidental, and due to planned human activities, is the main cause of soil pollution [9]. Being aggressive agents, petroleum hydrocarbons, once released into the environment, can alter the activity and efficiency of ecosystems and even pose a threat to human health [10,11]. As they are hardly degradable and highly toxic, petroleum hydrocarbons are persistent and hazardous organic pollutants [12]. Hydrocarbons show carcinogenic effects, limit the supply of major nutrients, such as nitrogen and phosphorus [13], raise the hydrophobicity of soil and increase the emission of CO_2_ [14], in addition to which they change the chemical composition of plants [15,16] and contribute to chlorosis and necrosis of plant tissues [17,18].

The number of potentially polluted sites in the European Union is estimated at 2.5 million, of which 342,000 have had the origin of contamination identified. The prevalent pollutants are diesel oil and trace elements [19]. Because of the scale of this problem and global ecological policies, reclamation of polluted soils, including the search for novel technologies, is an issue attracting growing interest and greater need for studies.

Waste from incineration processes, especially fly ash from conventional heat and power generation or from thermal conversion of sewage sludge, is a by-product that is difficult to dispose of and recycle [20,21]. In accordance with the principles of sustainable development, it is harmful for the environment to dispose of such waste on landfills [22]. Ash from heat and power plants, fired with coal or ash produced during the recycling of sewage sludge, is treated as waste containing some elements useful in agricultural production (Ca, Mg, P, Fe, Zn, Mo, Mn, Cu) [23,24] and, therefore, can be added to soil to improve some of its physicochemical properties (e.g., higher sorption capacity, higher base saturation, better water relations and lower acidity), especially in the case of light oils [20,25]. Because of its chemical composition, such ash can also be treated as a substitute or ingredient of mineral fertilizers [26,27]. Supplementation of soil with waste ash can improve the balance of nutrients in the environment [21,28,29] and limit the adverse effect of its excessive concentration in the vicinity of landfills or heaps. Ash shows a positive effect on the properties and yield of plants [30,31,32], including energy crops [22,33], and on reclamation of degraded lands [30,34,35]. By using waste ash for fertilization purposes, we satisfy some principles of sustainable growth, mainly the preservation and closed circulation of nutrients in an entire ecosystem.

It is not sufficient to set up energy crop plantations to enlarge the share of biomass in energy generation [36]. While it is necessary to manage degraded lands for this purpose, eco-friendly methods for the reclamation of such soils need to be developed. At the same time, particular attention should be paid to the proper selection of plants to reduce pollutants, which are a large threat to the environment, but also reduce agricultural productivity of soils [37,38,39]. Further, to detoxify soil polluted with petroleum products, it is necessary to search for various materials that chelate hydrocarbons and substances that improve the oxygenation of soils. This treatment can speed up the degradation of hydrocarbons [40,41]. A faster degradation of petroleum products will positively affect the improvement of the chemical composition of plants [42,43]. It seems that such a versatile approach can provide the global community with energy-related, economic and environmental benefits. Furthermore, efficient recycling of various forms of ash for the reclamation of degraded soils, which could then be used for growing energy crops, would correspond well with the concept of sustainable development and environmental protection, broadly understood.

In the current study, we hypothesized that the application of ashes (coal ash and sewage sludge ash) to the soil would limit the negative impact of Eco-Diesel oil on biomass and the content of macronutrients in maize (*Zea mays* L.). This led to detailed predictions that: (1) Eco-Diesel oil impact on the biomass of maize would be negative, with increased content of macronutrients in the aerial parts of maize, (2) the application of ashes to the soil would limit the effect of Eco-Diesel oil on plants, (3) sewage sludge ash would have a greater effect than coal ash on the content of macronutrients and biomass of maize.

## 2. Materials and Methods

### 2.1. Design of the Experiment

A plant-growing pot experiment was carried out in a greenhouse at the University of Warmia and Mazury in Olsztyn (north-eastern Poland), using soil which, according to the soil taxonomy by the International Union of Soil Sciences and the United States Department of Agriculture [44], had the texture of sandy loam (sand >0.05 mm, 51.20%; silt 0.02–0.05 mm, 45.34%; and clay <0.002 mm, 1.46%). Polyethylene pots were each filled with 2.8 kg of soil (classified as eutric cambisol) contaminated with increasing doses of diesel oil, brand name Eco-Diesel: 0, 10 and 20 cm^3^ kg^−1^ dry matter (d.m.) of soil. The substance applied to alleviate the contamination was ash from incineration of hard coal and ash from the burning of sewage sludge, in amounts of 0, 0.25, 0.5, 1 and 2 g kg^−1^ of soil. Therefore, the following substrates were used in the experiment: (1) uncontaminated soil, (2) soil contaminated with Eco-Diesel oil (ED), (3) uncontaminated soil with the addition of coal ash, (4) soil contaminated with ED with the addition of coal ash, (5) uncontaminated soil with the addition of sewage sludge ash, and (6) soil contaminated with ED with the addition of sewage sludge ash. The physicochemical composition of the soil and ash used in the experiment is presented in Table 1. Additionally, soil in each pot was mixed with macronutrients in the following doses and forms: nitrogen, 112 mg [CO(NH_2_)_2_]; phosphorus, 39 mg [KH_2_PO_4_]; potassium, 112 mg [KH_2_PO_4_ + KCl]; magnesium, 15 mg kg^−1^; and sulfur, 19.8 mg d.m. of soil [MgSO_4_·7H_2_O]. The test plant was maize (*Zea mays* L.) of the variety LG 32.58 (variety registered in European Union), with six plants seeded in every pot, and each experimental variant set up in 4 replications. The soil moisture was maintained at a constant level (60% of the capillary water capacity) throughout the entire experiment (60 days). Maize was harvested after the emergence of panicles (BBCH 59) for collecting plant samples for laboratory analyses.

### 2.2. Research Procedures

Samples of the aerial parts of maize plants were cut, dried and ground. Then, the plant samples were wet-ashed in concentrated 96% sulfuric acid with the addition of 30% H_2_O_2_ [45]. The mineralized samples underwent the following determinations: content of total nitrogen, by Kjeldahl’s method [46]; phosphorus, by colorimetry [45]; and potassium, calcium, magnesium and sodium, by atomic absorption/emission spectrometry ASA [45] on a SpectrAA 240FS spectrophotometer apparatus (Varian Inc., Mulgrave, VIC, Australia).

The following soil properties were analyzed before setting up the experiment: The areometric method was used to determine the granulometric composition of soil [47]. This is a method belonging to a group of sedimentation methods. The shaded soil is sieved through a sieve with a diameter of 2 mm meshes to separate the skeleton parts from the soil part. Then the soil parts are analyzed using the areometer. Determination of granulometric composition consists in measuring the soil slurry density during sedimentation of soil particles at a constant temperature. The other soil properties were analyzed as follows: pH in 1 M KCl with the potentiometric method [48], hydrolytic acidity and sum of exchangeable base cations with Kappen’s method [49], content of total nitrogen with Kjeldahl’s method [50], organic carbon with Tiurin’s method [51], available phosphorus and potassium with Egner–Riehm’s method [52] and available magnesium with Schachtschabel’s method [53].

### 2.3. Statistical Analysis

The results were processed statistically in a Statistica [54] package (TIBCO Software Inc., Palo Alto, CA, USA), by calculating the following: two-way analysis of variance (ANOVA), Pearson’s simple correlation coefficients, principal component analysis (PCA), percentage of observed variation using the η2 coefficient and the ANOVA method.

## 3. Results

### 3.1. The Effect of Eco-Diesel Oil Contamination on Maize

**Figure 1:**
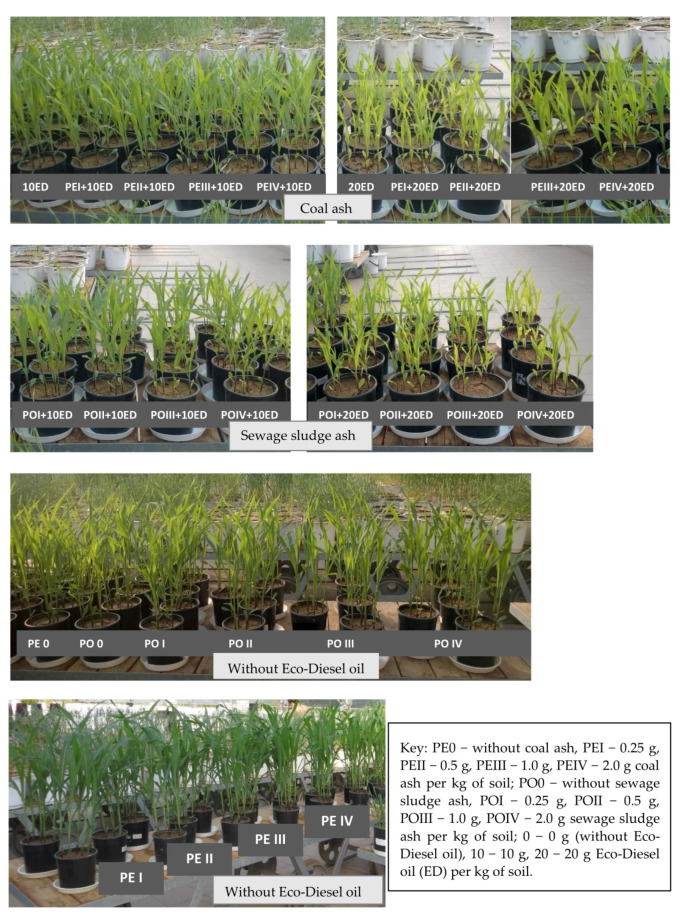
Pot experiment conducted in a greenhouse in the leaf development phase of maize-*Zea mays* L. (6–7 leaves, BBCH 16–17).

Maize growth and development was influenced more by Eco-Diesel oil than by ash. Maize plants grown on the soil polluted with a higher dose of ED were fragile and had signs of chlorosis (Figure 1). A dose of 10 cm^3^ ED kg^−1^ d.m. of soil caused a reduction of plant yield by 61%, and a dose of 20 cm^3^ ED kg^−1^ d.m. of soil by as much as 87% (Table 2). The content of macronutrients in the aerial biomass of maize was correlated with the doses of Eco-Diesel oil and the doses of the remediation substances (Table 3 and Table 4). The contamination of soil with diesel oil had a significant effect on the content of macronutrients, especially on the accumulation of phosphorus, potassium and calcium in the aerial organs of maize. The biggest changes were observed for calcium. In the series without added ash and treated with the highest dose of diesel oil (20 cm^3^ kg^−1^ of soil), the content of this element in the aerial parts of maize was twice as high as in the control (not polluted with diesel oil). No significant effect of the pollution was noticed only in the case of magnesium.

In the series without the remediation substances, soil contamination with increasing doses of the pollutant caused a decrease in the content of nitrogen and sodium in the maize aerial parts, by 8.3% and 15.2%, respectively, in comparison to the control. However, these changes were not significant statistically.

### 3.2. The Effect of Ashes Application on Maize on Soil Contaminated with Eco-Diesel Oil

The effect of ashes on the growth of plants grown in unpolluted soil was comparable. In the case of soil contaminated with a higher dose of Eco-Diesel oil (20 cm^3^ kg^−1^ d.m. of soil), a mitigating effect of ashes on maize was observed in objects supplemented with coal ash in the amount of 0.25 g kg^−1^ d.m. of soil and sewage sludge ash in the amount of 0.25 and 0.5 g kg^−1^ d.m. of soil (Figure 1, Table 2). The study has demonstrated that the soil incorporation of ash affected the chemical composition of maize (Table 3 and Table 4). When hard coal ash was added to soil in a dose of 2 g kg^−1^ d.m. of soil, a decrease in the content of phosphorus (by 12.6%) and potassium (by 2.6%) in the aerial parts of maize was determined compared to the control. However, no significant changes in the content of sodium, calcium and magnesium in the aerial organs of maize were noted. The soil application of this type of ash resulted in a varied increase in the content of nitrogen, depending on the dose of ash. The most beneficial effect (an increase by 8.8%) was recorded when the lowest dose of hard coal ash, i.e., 0.25 g kg^−1^ d.m. of soil, was tested.

The soil remediation with ash from sewage sludge incineration favored the accumulation of nitrogen and calcium in the maize aerial parts, while lowering the accumulation of phosphorus versus the control. The application of the highest tested dose of this ash (2 g kg^−1^ d.m. of soil) resulted in an 11.4% increase in the content of calcium compared to the control sample. In turn, the plant content of nitrogen changed the most in response (an 10.2% increase) to the ash dose of 0.5 g kg^−1^ d.m. of soil. However, no significant effect of this type of ash was determined with respect to potassium, sodium and magnesium in maize.

**Figure 2:**
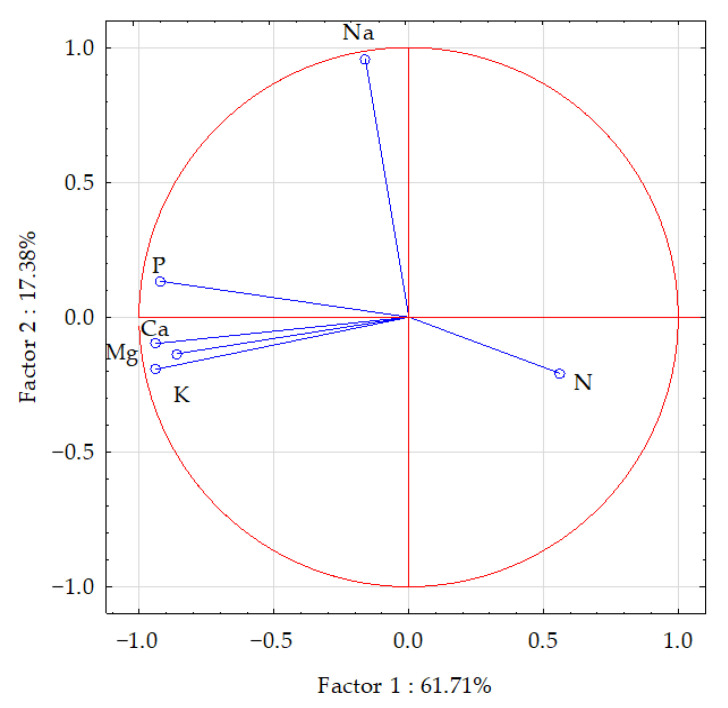
Content of macroelements in the aerial parts of maize (*Zea mays* L.) illustrated with the PCA method. Key: vectors represent analyzed variables (content of N, P, K, Na, Ca and Mg).

**Figure 3:**
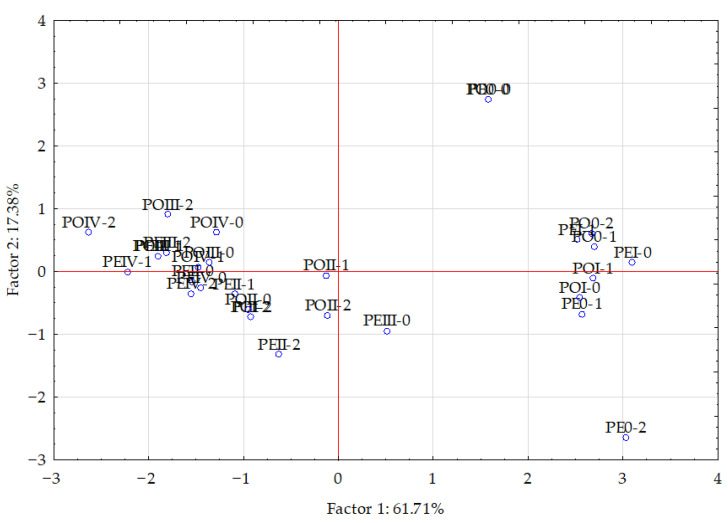
Effect of amendments on content of macroelements in the aerial parts of maize (*Zea mays* L.) illustrated with the PCA method. Key: points show the samples with elements (PE0—without coal ash, PEI—0.25 g, PEII—0.5 g, PEIII—1.0 g, PEIV—2.0 g; ash per kg of soil; PO0—without sewage sludge ash, POI—0.25 g, POII—0.5 g, POIII—1.0 g, POIV—2.0 g ash per kg of soil; 0—0 g (control), 1—10 g, 2—20 g eco-diesel oil per kg of soil.

The statistical analysis of the results (using PCA and Pearson’s linear correlation coefficient) showed a cumulative effect of soil pollution with Eco-Diesel oil and the application of the soil remediation agents on the content of macronutrients in the aerial parts of maize, which is illustrated by the correlation coefficients in Table 5 and vector variables in Figure 2. The PCA revealed that the principal components explained 61.71% of the input data variation, dividing the data into two groups (the first, phosphorus, potassium, calcium and magnesium; the second, nitrogen and sodium). Vectors of most of the analyzed elements were similar in length, except for nitrogen and magnesium, which were shorter, indicating their smaller contribution to the variation. The strongest positive correlation appeared between calcium versus potassium and phosphorus, and between phosphorus versus calcium and potassium. The strongest negative correlation appeared between nitrogen versus phosphorus, potassium, calcium and magnesium. Dispersion of the points in Figure 3 suggested that the application of the soil remediation agents tended to have a positive influence on the content of the analyzed elements in the aerial parts of maize.

**Figure 4:**
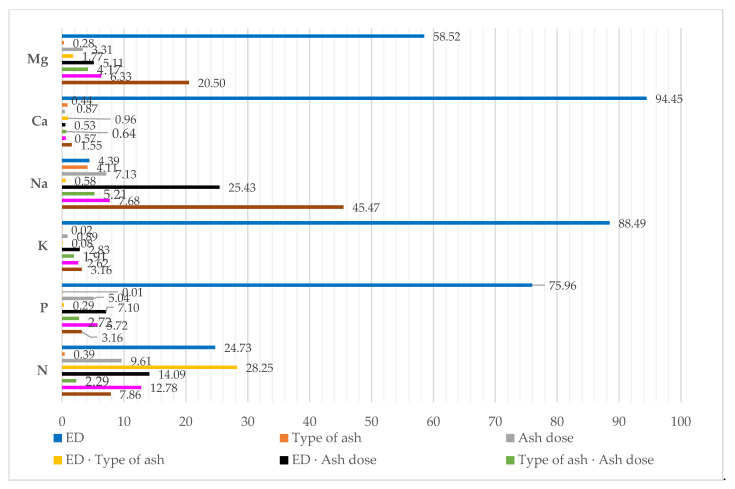
Percent contribution of variable factors according to the content of macroelements in aerial parts of maize (*Zea mays* L.): ED—Eco-diesel oil dose.

The percentage of the observed variation identified with η2, using the ANOVA method, showed that the content of macronutrients in maize was mostly affected by the dose of diesel oil polluting the soil. As for calcium, potassium, phosphorus and magnesium, this factor was responsible for 94.45%, 88.49%, 75.96% and 58.52% of the variation of the respective variables (Figure 4). Much smaller values were obtained for the other elements, i.e., sodium and nitrogen. They were 4.39% and 24.73%, respectively. The effect of the type and doses of ash on the chemical composition of aerial parts of maize was demonstrably weaker and never exceeded 10%, while being the strongest for a dose of ash and the content of nitrogen.

## 4. Discussion

### 4.1. The Effect of Eco-Diesel Oil Contamination on Plants

Plant biomass, which is widely available, renewable and relatively inexpensive to acquire, is a promising raw material to be used for energy purposes [55]. Maize is one of the target plant species with high yielding potential, particularly for production of high-quality biofuels [56,57]. It is distinguished by its high productivity of green mass per unit area [36,58], high efficiency of photosynthesis (1.5–2 times more efficient than that of C3 plants), moderate soil requirements and high adaptability to changing weather conditions [3,36]. The necessity to protect nature, limited resources of fossil fuel and increasing prices of petroleum require the development of alternative energy generation solutions [59].

Diesel oil, made from petroleum, is one of the most widespread soil contaminants [60]. Chemically, it is a mixture of saturated (e.g., paraffin, naphthenic) and aromatic (e.g., naphthalene and its derivatives, toluene, anthracene and phenanthrene) hydrocarbons [61]. Once they have entered soil, hydrocarbons, especially polycyclic aromatic hydrocarbons, have an adverse impact on the soil microbiome [62,63] and yield of different plant species [64,65,66], and, in high quantities, they can inhibit the germination of plants and induce necrosis of seedlings [67]. Additionally, they limit the soil capacity for exchange of calcium, magnesium and potassium, thereby lowering the availability of macronutrients and modifying their concentrations in particular plant organs [68,69]. In this study, increasing doses of Eco-Diesel oil added to soil resulted in the maize plants increasing the accumulation of phosphorus, potassium and calcium, while decreasing that of sodium and nitrogen. Similar dependences were demonstrated by Wyszkowski et al. [15], who applied diesel oil in a dose of 15 cm^3^ kg^−1^ d.m. of soil and observed an increase in the phosphorus and calcium content of aerial parts of oat by 38% and 34%, respectively, relative to the control. A positive correlation between soil pollution with diesel oil and the content of potassium in aerial parts of winter wheat has also been determined by Rusin et al. [70]. Wyszkowski and Ziółkowska [71] observed that diesel oil caused a rise in the content of available forms of potassium and magnesium in soil, which can explain their elevated levels in aerial parts of maize noted in this experiment. An analogous tendency has been recorded by Borowik and Wyszkowska [66] in their experiment, where the content of available phosphorus and magnesium, as well as exchangeable magnesium and potassium, increased in soil contaminated with a dose of diesel oil equal to 10 cm^3^ kg^−1^.

The decreasing content of nitrogen in aerial parts of maize observed in this experiment had been previously reported by Wyszkowski and Wyszkowska [72] to have occurred in oat biomass harvested from soil contaminated with 24 g of diesel oil per kg of soil. In the same experiment, but in a parallel series, diesel oil favored the accumulation of most macronutrients in the aerial parts of maize. The contamination of soil with petroleum substances leads to some disturbance in the carbon to nitrogen ratio [73], which may cause a change in the course of nitrogen transformations in soil, affecting the intensity of ammonification and nitrification processes [74,75]. In consequence, soil nitrogen losses occur [73,74], and the phytoavailability of this element suffers, so that, eventually, the content of nitrogen in plant biomass is reduced.

### 4.2. The Effect of Ashes Application on Plants on Soil Contaminated with Eco-Diesel Oil

The application of ash to soil may preferentially affect the yield of maize. According to Gao and DeLuca [76], ash application into the soil has a positive impact on the growth and development of plants and increases their yield and nutrient uptake by plants. In studies by Hale et al. [77] the effect of ashes on maize yield was stronger than biochar and lime. In their studies, ash caused a 9-fold increase in the yield of maize. According to Saletnik et al. [78], a favorable effect on the yield of plants was smaller, though positive (maximum increase in yield of 68%). A beneficial effect of ashes on maize yield was confirmed in our own studies, however, their impact was smaller than those obtained in other authors’ experiments [77,78]. Too high doses of ashes do not increase, and can even have a negative effect on, the yield of plants [79].

The enrichment of soil with ash differentiated the chemical composition of maize in our experiment. Fly ash from the heat and power plant stimulated the accumulation of nitrogen while decreasing the concentrations of phosphorus and potassium in the aerial parts of the test plant. A similar effect of fly ash on the content of phosphorus in maize aerial organs has been noted by Antonkiewicz [80], who determined the highest coefficient of variation for magnesium (V = 32.55%), and the lowest for potassium (V = 18.40%). An increasing content of magnesium, calcium and phosphorus, as well as a decreasing content of potassium, in shoots of lucerne with increasing doses of fly ash (0–40%), were demonstrated by He et al. [21]. The differences in results may have been a consequence of the applied soil (lessivé soil) and doses of ash used in the cited experiments. The enrichment of soil with fly ash in a study conducted by Padhy et al. [81] raised the content of potassium and phosphorus in rice grain, but did not affect the level of sodium, which partly agrees with our results. In their 6-year field experiment, Antonkiewicz et al. [82] noted a decreased content of phosphorus in the biomass of a mixture of grasses and legumes, grown on soil enriched with fly ash in doses of 5–10 kg m^−2^. The reduced accumulation of phosphorus is likely to be associated with the occurrence of this element in poorly phytoavailable forms, e.g., calcium phosphates or hydroxyapatites, in soils enriched with hard coal fly ash [83]. Additionally, Li et al. [84] state that fly ash can bind phosphorus in soil, thereby diminishing its uptake by plants.

Despite the small content of nitrogen in fly ash (0–0.2%) [85], the uptake of this element by plants is higher in soils enriched with ash material. This is probably caused by the stimulating effect of ash on the mineralization of organic matter, which increases the pool of inorganic nitrogen available to plants [86].

The fly ash used in this experiment originating from the incineration of sewage sludge contributed to an increase in the content of nitrogen and calcium in the aerial biomass of maize. It limited the accumulation of phosphorus, while having no effect on the plant content of the remaining macronutrients submitted to analysis. Increase in the accumulation of nitrogen (14.7%) and calcium (8.6%) in maize growing on soil previously treated with ash from thermal conversion of sewage sludge was also reported by Iżewska and Wołoszyk [87]. In that experiment, the highest dose of fly ash (65.40 kg ha^−1^) resulted in an increase in the content of phosphorus and calcium in grain by 33.0% and 33.6%, respectively, compared to the series treated with mineral fertilizers (NPK). Zalewska et al. [88] concluded that fly ash obtained from incineration of sewage sludge is a good source of available phosphorus, magnesium and potassium in maize cultivation. Its soil application resulted in an over 5-fold higher yield relative to the control, and an increased uptake of the mentioned elements by the test plant. However, the content of macronutrients in the maize biomass from the control series was higher than in the other experimental variants, which the authors suggested was a consequence of a very small yield in the control series and subsequent conversion of the results per dry matter.

Pidlisnyuk et al. [89] evaluated the possibility of growing a perennial energy crop, such as *Miscanthus giganteus*, on land polluted with diesel oil (in a range from 250 mg kg^−1^ to 5000 mg kg^−1^). In response to increasing doses of the pollutant, these researchers observed a lower height of the plants and a smaller number of leaves. The application of sewage sludge biochar, wood waste and biohumus to soil enriched the soil’s pool of nutrients, extended the plant growing period and improved the plants’ morphological and physiological traits, alleviating the consequences of the abiotic stress. An addition of biochar from sewage sludge enabled the *Miscanthus giganteus* plant to achieve the highest biomass productivity during harvest. According to these authors, the use of biochar can be an effective method of reclamation of soil contaminated with diesel oil, so that such soil can be then used for growing crops with an alternative use, such as production of fuels.

Maize is one of the most important cultivated plants in the world. It constitutes 12% (second place) of global production of cultivated plants [90]. Production of maize in recent years has increased and, according to forecasts, will grow in subsequent years (by 10% in 2030 compared to 2018–2020) [91]. The results obtained in our own studies, since the test plant was maize, which is grown on all continents, can be used in every area of the world.

## 5. Conclusions

The yield and chemical composition of maize depended on the degree of soil pollution with Eco-Diesel oil and on soil enrichment with neutralizing substances. Eco-Diesel oil had a negative effect on maize yield. A positive relationship was demonstrated between increasing doses of Eco-Diesel oil and the accumulation of all macronutrients in the aerial parts of maize, except nitrogen and sodium. The enrichment of soil with fly ash increased maize biomass and the content of some macronutrients, especially nitrogen, and calcium, the latter in the series where soil was treated with fly ash from the thermal conversion of sewage sludge. Additionally, both types of fly ash resulted in a decreased content of phosphorus, while hard coal fly ash led to a small reduction in the content of potassium in maize. 

Fly ash of different origins can be an effective instrument for reclamation of degraded soils intended for growing energy crops. The positive results of our own research were obtained with maize, which is one of the most important plants in the world used for production of feed and food. The maize-growing area in the world allows for the managed use of ashes, as a waste material, on a large scale.

## Figures and Tables

**Table 1:** Physicochemical parameters of soil and ashes used in research.

Physicochemical Parameters	Unit	Soil	Coal Ash	Sewage Sludge Ash
pH in 1 M KCl		6.73		
Hydrolytic acidity	mM_(+)_ kg^−1^	8.0		
Cation exchange capacity (CEC)		126.7		
Total exchangeable bases (TEB)		134.7		
Base saturation (BS)	%	94.6		
Organic carbon (TOC)	g kg^−1^ d.m.	12.60		
Total nitrogen		1.08	1.429	4.638
Total phosphorus			10.20	88.11
Total potassium			1.408	12.833
Total calcium			2.492	16.832
Total magnesium			10.04	14.60
Total sodium			3.332	1.722
Available phosphorus	mg kg^−1^ d.m.	83.28		
Available potassium		178.50		
Available magnesium		3.20		

**Table 2:** Yield of aerial mass of maize-*Zea mays* L., g fresh weight (f.w.) pot^−1^.

Eco-Diesel Oil Dose (cm^3^ kg^−1^ d.m. of Soil)	Ash Dose (g kg^−1^ d.m. of Soil)					Average
	0	0.25	0.5	1	2	
Coal ash						
0	283.02 *^d^*	295.26 *^c^*	316.13 *^b^*	292.57 *^cd^*	291.17 *^cd^*	295.63 *^B^*
10	111.29 *^ef^*	122.08 *^e^*	113.31 *^ef^*	113.54 *^ef^*	120.88 *^e^*	116.22 *^C^*
20	36.03 *^ghij^*	42.14 *^ghij^*	32.44 *^j^*	31.20 *^j^*	33.70 *^ij^*	35.10 *^F^*
Average	143.45 *^V^*	153.16 *^II,III,IV^*	153.96 *^II,III,IV^*	145.77 *^V^*	148.58 *^III,IV,V^*	148.98
Sewage sludge ash						
0	283.02 *^d^*	311.65 *^b^*	291.38 *^cd^*	317.18 *^b^*	334.76 *^a^*	307.60 *^A^*
10	111.29 *^ef^*	104.89 *^f^*	103.60 *^f^*	107.49 *^f^*	112.74 *^ef^*	108.00 *^D^*
20	36.03 *^ghij^*	46.72 *^gh^*	47.63 *^g^*	44.67 *^ghi^*	39.99 *^ghij^*	43.01 *^E^*
Average	143.45 *^V^*	154.42 *^II,III^*	147.54 *^IV,V^*	156.44 *^II^*	162.50 *^I^*	152.87

Values denoted by the different letters and Roman numbers are significantly different at *p* ≤ 0.01: *^A–E^* for Eco-Diesel oil dose, *^I–V^* for ash dose and *^a–j^* for interaction between Eco-Diesel oil dose and ash dose (Anova, Tukey’s HSD test).

**Table 3:** Content of total-N (N), phosphorus (P) and potassium (K) in aerial parts of maize-*Zea mays* L. (g kg^−1^ d.m.).

Eco-Diesel Oil Dose (cm^3^ kg^−1^ d.m. of Soil)	Ash Dose (g kg^−1^ d.m. of Soil)					Average
	0	0.25	0.5	1	2	
Total-N (N)						
Coal ash						
0	6.720 *^abc^*	8.120 *^d^*	7.420 *^cd^*	7.140 *^bcd^*	6.720 *^abc^*	7.224 *^A^*
10	6.300 *^abc^*	5.880 *^a^*	6.300 *^abc^*	6.300 *^abc^*	6.580 *^abc^*	6.272 *^B^*
20	6.160 *^ab^*	6.860 *^abc^*	6.580 *^abc^*	6.580 *^abc^*	7.140 *^bcd^*	6.664 *^C^*
Average	6.393 *^I^*	6.953 *^II^*	6.767 *^I,II^*	6.673 *^I,II^*	6.813 *^I,II^*	6.720
Sewage sludge ash						
0	6.720 *^abcde^*	7.140 *^bcde^*	7.280 *^cde^*	7.140 *^bcde^*	6.860 *^abcde^*	7.028 *^A^*
10	6.300 *^abc^*	7.280 *^cde^*	7.840 *^e^*	7.700 *^de^*	7.000 *^abcde^*	7.224 *^A^*
20	6.160 *^abc^*	5.880 *^a^*	6.020 *^ab^*	6.020 *^ab^*	6.580 *^abcd^*	6.132 *^B^*
Average	6.393 *^I^*	6.767 *^I,II^*	7.047 *^II^*	6.953 *^II^*	6.813 *^I,II^*	6.795
Phosphorus (P)						
Coal ash						
0	2.409 *^cde^*	2.426 *^cde^*	1.679 *^a^*	1.831 *^ab^*	1.877 *^abc^*	2.044 *^A^*
10	2.714 *^defg^*	2.744 *^defg^*	2.649 *^defg^*	2.472 *^def^*	2.314 *^bcd^*	2.579 *^B^*
20	3.034 *^fg^*	2.758 *^defg^*	3.173 *^g^*	3.065 *^g^*	2.937 *^efg^*	2.993 *^C^*
Average	2.719 *^III^*	2.643 *^II,III^*	2.500 *^I,II,III^*	2.456 *^I,II^*	2.376 *^I^*	2.539
Sewage sludge ash						
0	2.409 *^abcd^*	1.959 *^a^*	1.997 *^ab^*	2.214 *^abc^*	1.967 *^a^*	2.109 *^A^*
10	2.714 *^de^*	2.622 *^cde^*	2.459 *^bcd^*	2.543 *^cd^*	2.551 *^cd^*	2.578 *^B^*
20	3.034 *^ef^*	2.817 *^def^*	2.765 *^de^*	2.844 *^def^*	3.282 *^f^*	2.948 *^C^*
Average	2.719 *^II^*	2.466 *^I^*	2.407 *^I^*	2.534 *^I,II^*	2.600 *^I,II^*	2.545
Potassium (K)						
Coal ash						
0	11.48 *^ab^*	12.75 *^ab^*	11.30 *^ab^*	10.68 *^a^*	10.65 *^a^*	11.37 *^A^*
10	16.42 *^cd^*	16.57 *^cd^*	17.60 *^cd^*	19.45 *^d^*	14.67 *^bc^*	16.94 *^B^*
20	17.18 *^cd^*	17.47 *^cd^*	17.26 *^cd^*	18.93 *^d^*	18.59 *^d^*	17.88 *^B^*
Average	15.03 *^I,II^*	15.60 *^I,II^*	15.39 *^I,II^*	16.35 *^II^*	14.64 *^I^*	15.40
Sewage sludge ash						
0	11.48 *^a^*	11.61 *^a^*	11.57 *^a^*	10.95 *^a^*	10.94 *^a^*	11.31 *^A^*
10	16.42 *^b^*	18.47 *^b^*	16.91 *^b^*	16.38 *^b^*	17.06 *^b^*	17.05 *^B^*
20	17.18 *^b^*	17.83 *^b^*	17.61 *^b^*	16.79 *^b^*	18.44 *^b^*	17.57 *^B^*
Average	15.03 *^I^*	15.97 *^I^*	15.36 *^I^*	14.71 *^I^*	15.48 *^I^*	15.31

Values denoted by the different letters and Roman numbers are significantly different at *p* ≤ 0.01: *^A–C^* for Eco-Diesel oil dose, *^I^*^–*III*^ for ash dose and *^a^*^–*g*^ for interaction between Eco-Diesel oil dose and ash dose (Anova, Tukey’s HSD test).

**Table 4:** Content of sodium (Na), calcium (Ca) and magnesium (Mg) in aerial parts of maize-*Zea mays* L. (g kg^−1^ d.m.).

Eco-Diesel Oil Dose (cm^3^ kg^−1^ d.m. of Soil)	Ash Dose (g kg^−1^ d.m. of Soil)					Average
	0	0.25	0.5	1	2	
Sodium (Na)						
Coal ash						
0	0.934 *^a^*	0.738 *^a^*	0.607 *^a^*	0.772 *^a^*	0.792 *^a^*	0.768 *^A^*
10	0.730 *^a^*	0.764 *^a^*	0.761 *^a^*	0.699 *^a^*	0.712 *^a^*	0.734 *^A^*
20	0.792 *^a^*	0.828 *^a^*	0.758 *^a^*	0.795 *^a^*	0.783 *^a^*	0.791 *^A^*
Average	0.819 *^I^*	0.777 *^I^*	0.709 *^I^*	0.756 *^I^*	0.762 *^I^*	0.764
Sewage sludge ash						
0	0.934 *^b^*	0.791 *^ab^*	0.809 *^ab^*	0.730 *^a^*	0.748 *^ab^*	0.803 *^A^*
10	0.730 *^a^*	0.774 *^ab^*	0.818 *^ab^*	0.768 *^ab^*	0.824 *^ab^*	0.783 *^A^*
20	0.792 *^ab^*	0.834 *^ab^*	0.813 *^ab^*	0.775 *^ab^*	0.834 *^ab^*	0.809 *^A^*
Average	0.819 *^I^*	0.800 *^I^*	0.813 *^I^*	0.758 *^I^*	0.802 *^I^*	0.798
Calcium (Ca)						
Coal ash						
0	7.778 *^a^*	7.359 *^a^*	8.790 *^ab^*	7.522 *^a^*	7.581 *^a^*	7.806 *^A^*
10	12.272 *^c^*	12.331 *^cd^*	12.252 *^c^*	11.350 *^bc^*	12.346 *^cd^*	12.110 *^B^*
20	15.467 *^de^*	15.926 *^e^*	15.645 *^e^*	16.108 *^e^*	15.614 *^e^*	15.752 *^C^*
Average	11.839 *^I^*	11.872 *^I^*	12.229 *^I^*	11.660 *^I^*	11.847 *^I^*	11.889
Sewage sludge ash						
0	7.778 *^a^*	8.198 *^a^*	8.129 *^a^*	8.312 *^a^*	8.452 *^a^*	8.174 *^A^*
10	12.272 *^b^*	14.089 *^bc^*	13.603 *^bc^*	13.890 *^bc^*	14.164 *^bc^*	13.604 *^B^*
20	15.467 *^cd^*	15.491 *^cd^*	15.027 *^cd^*	15.696 *^cd^*	16.956 *^d^*	15.727 *^C^*
Average	11.839 *^I^*	12.593 *^I,II^*	12.253 *^I,II^*	12.633 *^I,II^*	13.190 *^II^*	12.502
Magnesium (Mg)						
Coal ash						
0	1.995 *^a^*	1.899 *^a^*	2.017 *^a^*	1.859 *^a^*	2.007 *^a^*	1.955 *^A^*
10	2.378 *^a^*	2.528 *^a^*	2.391 *^a^*	2.210 *^a^*	2.105 *^a^*	2.322 *^B^*
20	2.312 *^a^*	2.521 *^a^*	2.112 *^a^*	2.372 *^a^*	2.319 *^a^*	2.327 *^B^*
Average	2.228 *^I^*	2.316 *^I^*	2.173 *^I^*	2.147 *^I^*	2.144 *^I^*	2.202
Sewage sludge ash						
0	1.995 *^a^*	1.850 *^a^*	1.875 *^a^*	1.866 *^a^*	1.842 *^a^*	1.886 *^A^*
10	2.378 *^a^*	2.361 *^a^*	2.314 *^a^*	2.278 *^a^*	2.620 *^a^*	2.390 *^B^*
20	2.312 *^a^*	2.234 *^a^*	2.102 *^a^*	2.200 *^a^*	2.402 *^a^*	2.250 *^B^*
Average	2.228 *^I^*	2.148 *^I^*	2.097 *^I^*	2.115 *^I^*	2.288 *^I^*	2.175

Values denoted by the different letters and Roman numbers are significantly different at *p* ≤ 0.01: *^A^*^–*C*^ for Eco-Diesel oil dose, *^I^*^–*III*^ for ash dose and *^a^*^–*g*^ for interaction between Eco-Diesel oil dose and ash dose (Anova, Tukey’s HSD test).

**Table 5:** Correlation coefficients (r) between content of macroelements in aerial parts of maize (*Zea mays* L.).

Macroelements	P	K	Na	Ca	Mg
N	−0.477 **	−0.397 **	−0.114	−0.407 **	−0.342 **
P		0.834 **	0.258	0.862 **	0.683 **
K			−0.001	0.903 **	0.821 **
Na				0.08	0.07
Ca					0.765 **

Significant at ** *p* ≤ 0.01.

## Data Availability

Data are available by contacting the authors.
